# How to mitigate selection bias in COVID-19 surveys: evidence from five national cohorts

**DOI:** 10.1007/s10654-024-01164-y

**Published:** 2024-11-20

**Authors:** Martina K. Narayanan, Brian Dodgeon, Michail Katsoulis, George B. Ploubidis, Richard J. Silverwood

**Affiliations:** 1 Centre for Longitudinal Studies, UCL Social Research Institute, University College London, London, UK; 2 MRC Unit for Lifelong Health & Ageing, University College London, London, UK

**Keywords:** COVID-19, Longitudinal data, Non-response, Missing data, Multiple imputation, Weighting

## Abstract

Non-response to surveys is a common problem; even more so during the COVID-19 pandemic with social distancing measures challenging data collection. As respondents often differ from non-respondents, this can introduce bias. The goal of the current study was to see if we can reduce bias and restore sample representativeness in a series of COVID-19 surveys embedded within five UK cohort studies by using the rich data available from previous waves of data collection. Three surveys were conducted during the pandemic across five UK cohorts: National Survey of Health and Development (NSHD, born 1946), 1958 National Child Development Study (NCDS), 1970 British Cohort Study (BCS70), Next Steps (born 1989-90) and Millennium Cohort Study (MCS, born 2000-02). Response rates in the COVID-19 surveys were lower compared to previous waves, especially in the younger cohorts. We identified bias due to systematic non-response in several variables, with more respondents in the most advantaged social class and among those with higher childhood cognitive ability. Making use of the rich data available pre-pandemic in these longitudinal studies, the application of non-response weights and multiple imputation was successful in reducing bias in parental social class and childhood cognitive ability, nearly eliminating it for the former. Surveys embedded within existing cohort studies offer a clear advantage over cross-sectional samples collected during the pandemic in terms of their ability to mitigate selection bias. This will enhance the quality and reliability of future research studying the medium and long-term effects of the pandemic.

## Introduction

A large amount of research studying the impact of the COVID-19 pandemic is based on web surveys, phone surveys and other selective samples recruited for the first time during the pandemic [1, 2]. These selective samples often lack representativeness of their target population because respondents differ systematically from non-respondents, introducing potential bias. Correcting this bias is challenging without information on non-respondents. Common correction methods include reweighting samples based on population distributions, but these may also be inadequate due to limited population information, particularly during the pandemic. Embedding COVID-19 surveys within existing longitudinal population-based studies offers an alternative by leveraging pre-pandemic data to mitigate bias due to selective response.

In this paper we aim to describe the response to the COVID-19 surveys embedded within five UK longitudinal cohort studies. We further detail the implementation of non-response weights and multiple imputation (MI) to handle missing data due to non-response, capitalising on the rich data cohort members provided prior to the COVID-19 surveys in order to restore sample representativeness. Showing that sample representativeness can be restored for these COVID-19 surveys is especially important for future research studying the medium and long-term effects of the pandemic. This work builds upon recent work on appropriately handling non-response in three of these cohorts [3–5].

## Methods

### Data

We used information from five nationally representative cohort studies, whose participants have been providing information about their lives since childhood. All cohorts were designed to be representative of their target population and response rates to the issued samples at initial data collection were high [6, 7]. NSHD, Next Steps and MCS provide design weights to further ensure representativeness [8]. Recent publications demonstrate that cohort estimates compare well with official population estimates at later time points for NCDS, BCS70 and Next Steps when using weights or multiple imputation [3–5]. Brief details of the studies are given here; full details are available elsewhere [6, 7].

#### National Survey of Health and Development (NSHD)

The NSHD is a representative sample (*N* = 5362) of men and women born in England, Scotland, and Wales in March 1946. Data were collected from birth and study members have been followed up 24 times. At the first wave of the COVID-19 survey cohort members were around 73 years old.

#### 1958 National Child Development Study (NCDS)

The NCDS is a representative sample of 17,500 babies born in England, Scotland, and Wales in one week of 1958. The birth survey has been followed by ten further data collections. At the first wave of the COVID-19 survey cohort members were around 62 years old.

#### 1970 British Cohort Study (BCS70)

The BCS70 is a representative sample of more than 17,000 people born in England, Scotland, and Wales in a single week of 1970. Following the birth survey there have so far been eight more surveys. At the first wave of the COVID-19 survey cohort members were around 50 years old.

#### Next Steps

Next Steps, previously known as the Longitudinal Study of Young People in England (LSYPE), follows the lives of around 16,000 people in England born in 1989-90. Next Steps was designed to be representative of young people in Year 9 at the time. Cohort members have been surveyed 8 times starting at age 14 years. At the first wave of the COVID-19 survey cohort members were around 31 years old.

#### Millennium Cohort Study (MCS)

The MCS is a nationally representative study following the lives of around 19,000 young people born across England, Scotland, Wales, and Northern Ireland in 2000-02. The first data collection took place at 9 months with six follow up surveys since then. At the first wave of the COVID-19 survey cohort members were around 20 years old.

#### COVID-19 surveys

A series of three surveys was conducted across all five cohorts during the pandemic [8]. A first COVID-19 survey (Wave 1) took place in May 2020 at the time when the UK was in a first national lockdown, with over 15,000 study participants taking part across the five cohorts. Nearly 20,000 participants took part in a second survey (Wave 2) in September/October 2020, during a period in which lockdown restrictions had been mostly lifted. The Wave 3 survey took place in February/March 2021, during the third UK lockdown, with over 22,000 participants.

The target population of each cohort is identified as cohort members who are alive and still residing in the UK to appropriately match the actual UK population. Information on mortality and emigration was not available for MCS and Next Steps, but rates of mortality and emigration are likely to be low in these cohorts.

### Measures

#### Covariates

Covariates included in the derivation of non-response weights and in imputation models are listed in Table 1. The choice of covariates was informed by previous work identifying important predictors of non-response in the British cohort studies, maximising the plausibility of the missing at random assumption [3–5]. More details on coding of all variables can be found in Narayanan et al. [9].

**Table 1:** Variables included in the weight derivation models and imputation models

	NSHD	NCDS	BCS70	Next Steps	MCS
Sex	Birth	Birth	Birth	Age 14	9 months
Ethnicity	-	-	-	Age 14	9 months Age 3
Parental social class	Age 4^G^ Age 11^F^	Birth Age 11^F^	Birth Age 10^F^	Age 14^F^	9 months Age 11^F^
Number of rooms at home/persons per room	Birth	Birth	Birth	-	9 months
Cognitive ability	Age 8^F^ Age 11	Age 7^F^ Age 11	Age 10 Age 5^F^	-	Age 5^F^ Age 7
Early life mental health	Age 13 & 15	Age 16	Age 16	Age 15	Age 11
Voting	Age 26	Age 42	Age 42	Age 20	NA
Membership in organisations	Age 43	Age 42	Age 42	Age 26	Age 14
Internet access prior to web survey	Age 69	Age 50	Age 46	Age 26	Age 14
Consent for biomarkers	Age 60-64^B^	Age 44	Age 46	-	-
Consent for linkages	Age 60-64^B^	-	-	Age 26	-
Educational qualifications	Age 26	Age 42	Age 42	Age 26	9 months^A^
Economic activity	Age 60–64	Age 50	Age 46	Age 26	Age 14^A^
Partnership status	Age 69	Age 50	Age 46	Age 26	Age 14
Psychological distress	Age 69	Age 50	Age 46	Age 26	Age 14
BMI	Age 69	Age 50	Age 46	Age 26	Age 11
Self-rated health	Age 69	Age 50	Age 46	Age 26	Age 14
Smoking status	Age 69	Age 50	Age 46	Age 26	Age 14
Maternal mental health^C^	-	-	-	-	9 months
Social capital/social support	Age 69	Age 50	Age 46	Age 26	Age 14
Income	Age 69	Age 55	Age 42	Age 26	Age 14^A^
Number of non-responses across all previous sweeps	Birth– age 69	Birth– age 55	Birth– age 42	Age 14– age 26	9 months– age 14
Response to COVID-19 Wave 1 survey^D^	Age 74	Age 62	Age 50	Age 30	Age 19
Response to COVID-19 Wave 2 survey^E^	-	Age 62	Age 50	Age 30	Age 19

^A^ Main respondent, > 90% mothers

^B^ Excluded from final model due to collinearity

^C^ Also available in BCS70 at age 16 but not included in model

^D^ Included in Wave 2 and 3 response models only

^E^ Included in Wave 3 response model only, apart from in NSHD where Wave 3 web survey was only issued to those who had responded to previous COVID-19 surveys

^F^ These were used as variables in the restoring sample representativeness examples, which means they were not included in the derivation of weights

^G^ Not included in multiple imputation model due to convergence issues

NSHD: National Survey of Health and Development; NCDS: 1958 National Child Development Study; BCS70: 1970 British Cohort Study; MCS: Millennium Cohort Study

#### Parental social class in childhood

The true distribution of parental social class is known, as the variable is observed in childhood in nearly all participants in each cohort. This serves as a comparator to examine potential bias and whether non-response weighting and MI can help correct that bias. In NSHD, NCDS and BCS parental social class was coded in three categories (professional/intermediate, skilled, and partly-/unskilled). For MCS it was a different three categories (managerial, intermediate, and routine/semi-routine). For Next Steps, it was four categories (managerial, intermediate, routine/semi-routine, and never worked). As all analyses were run separately for each cohort, we did not attempt to further harmonise this variable.

#### Childhood cognitive ability

Similar to parental social class, childhood cognitive ability measures are included to demonstrate how non-response weighting and MI can help restore representativeness. Cognitive ability was measured as a standardised score based on different subtests (NSHD: Reading Comprehension, Word Reading, Vocabulary and Picture Intelligence; NCDS: Southgate Group Reading Test, Copying Designs Test, Human Figure Drawing, Problem Arithmetic Test; BCS70: English Picture Vocabulary Test, Copying Designs Test, Human Figure Drawing; MCS: BAS II Naming Vocabulary, BAS II Pattern Construction, BAS II Picture Similarities). Next Steps does not have measures of childhood cognitive ability and was therefore not included.

### Statistical methods

#### Derivation of non-response weights

Non-response weights were derived for each cohort separately but following a common approach:

(1) modelling COVID-19 survey response conditional on a common set of covariates using logistic regression, (2) predicting the probability of response from the model, (3) calculating non-response weight as the inverse of the probability of response, (4) deciding whether truncation may be desirable, (5) calibrating non-response weights so that they sum to the number of COVID-19 survey respondents in each cohort. For further details of the derivation of weights see the COVID-19 Survey User Guide [8].

In some cases, the original non-response weights from the COVID-19 Survey User Guide [8] included the same measure of parental social class/childhood cognitive ability as our ‘restoring representativeness’ examples. For these specific cases, we created new non-response weights based on response models which did not include the particular variable of interest. We conducted sensitivity analyses providing estimates based on the original and the newly created non-response weights.

#### Multiple imputation (MI)

In parallel analyses, MI was conducted separately for each cohort to restore sample representativeness of parental social class and childhood cognitive ability. Imputation models included the variable of interest and all covariates also used in non-response weight derivation, ensuring comparability (see Table 1). Fifty imputed datasets were created using multiple imputation with chained equations (using linear regression for continuous, logistic regression for binary, ordinal logistic regression for ordinal and multinomial logistic regression for nominal variables).

#### Restoring sample representativeness

We examine whether non-response weights and MI can restore sample representativeness. For each wave of the COVID-19 survey, we compared the known distribution of parental social class (or childhood cognitive ability) across all cohort members to the distribution in COVID-19 survey respondents only (to assess bias) and in COVID-19 survey respondents after the application of the non-response weights or MI (to assess bias reduction). Design weights were included where applicable to account for survey structure [8].

All analyses were conducted using Stata version 18 (StataCorp LLC; College Station, TX).

## Results

### COVID-19 survey response

Response rates are presented in Table 2. The total response rates relative to the issued sample increased over time (37.5% in Wave 1, 39.1% in Wave 2 and 43.8% in Wave 3) and were strongly patterned by cohort/age within each wave (e.g. 68.3% for NSHD through to 26.6% for MCS in Wave 1). The total response rates of all cohort members with respect to the target population (20.8% in Wave 1, 27.7% in Wave 2 and 31.2% in Wave 3) were markedly lower than those with respect to the issued sample.

**Table 2:** COVID-19 Wave 1, 2 and 3 surveys: issued sample, target population and response by cohort

Wave 1				
Cohort	Issued sample	Response^A^within issued sample	Cohort members within target population^B^	Response within target population
NSHD	1,843	1,258 (68.3%)	3,758	1,170 (31.1%)
NCDS	8,943	5,178 (57.9%)	15,291	5,119 (33.5%)
BCS70	10,458	4,223 (40.4%)	17,486	4,132 (23.6%)
Next Steps	9,380	1,907 (20.3%)	15,770^C^	1,876 (11.9%)
MCS	9,946	2,645 (26.6%)	19,243	2,609 (13.6%)
Total	40,570	15,211 (37.5%)	71,548	14,906 (20.8%)
**Wave 2**				
NSHD	2,551	1,569 (61.5%)	3758	1,488 (39.6%)
NCDS	11,655	6,282 (53.9%)	15,291	6,228 (40.7%)
BCS70	12,133	5,320 (43.9%)	17,486	5,236 (29.9%)
Next Steps	11,529	3,664 (31.8%)	15,770^C^	3,609 (22.9%)
MCS	13,547	3,274 (24.2%)	19,243	3,233 (16.8%)
Total	51,415	20,109 (39.1%)	71,548	19,794 (27.7%)
**Wave 3**				
NSHD	1,559	1,399 (89.9%)	3,758	1,325 (35.3%)
NCDS	11,630	6,809 (58.5%)	15,291	6,757 (44.2%)
BCS70	12,683	5,758 (45.4%)	17,486	5,684 (32.5%)
Next Steps	12,349	4,239 (34.3%)	15,770^C^	4,167 (26.4%)
MCS	13,533	4,474 (33.1%)	19,243	4,422 (23.0%)
Total	51,574	22,679 (43.8%)	71,548	22,355 (31.2%)

^A^ Response was defined as completion of the first block of the questionnaire (“Physical health since outbreak”)

^B^ Those alive and still residing in the UK. Mortality and emigration data not available for Next Steps and MCS

^C^ Next Steps includes original sample only (i.e. not ethnic minority boost sample)

NSHD: National Survey of Health and Development; NCDS: 1958 National Child Development Study; BCS70: 1970 British Cohort Study; MCS: Millennium Cohort Study

### Restoring sample representativeness for parental social class

**Fig. 1:**
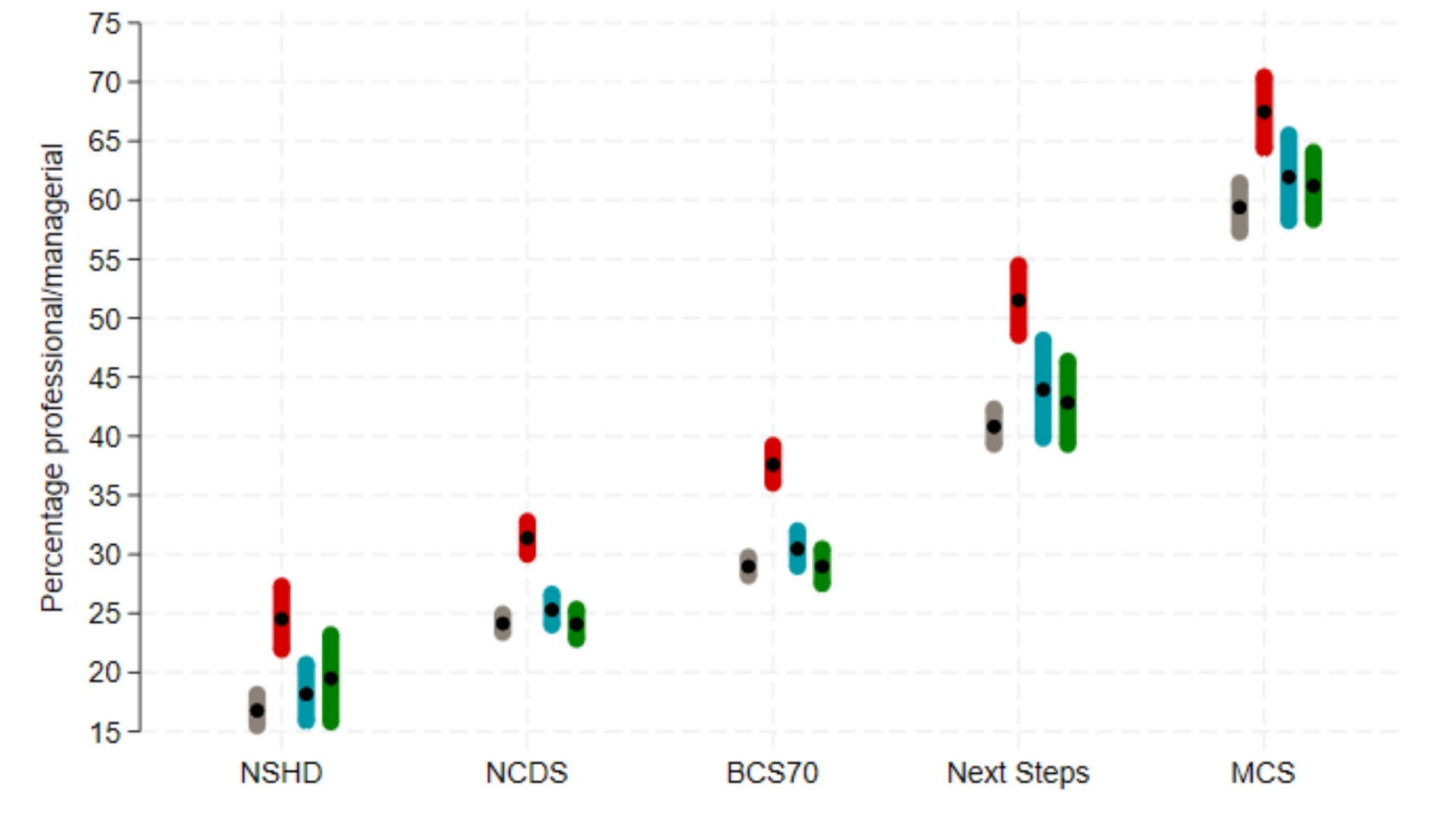
Percentage of highest social class (professional/managerial) in each cohort under different estimation approaches to account for non-response in the COVID-19 Wave 1 survey. Grey, first line: using observed baseline data from the whole cohort; red, second line: using observed baseline data from COVID-19 Wave 1 survey respondents only– unweighted (NCDS and BCS70) or using design weight only (NSHD, Next Steps and MCS); blue, third line: using observed baseline data from COVID-19 Wave 1 survey respondents only– weighted using non-response weights (in addition to design weights as appropriate); green, fourth line: using multiple imputation (plus design weight as appropriate). NSHD: National Survey of Health and Development; NCDS: 1958 National Child Development Study; BCS70: 1970 British Cohort Study; MCS: Millennium Cohort Study

Substantial bias was found in the estimated percentage of cohort members in the highest social class among COVID-19 survey respondents, with higher percentages from more advantaged social classes (see Fig. 1 for Wave 1). Non-response weights and MI reduced this bias, nearly eliminating it in most cohorts. Results for Wave 2 and 3 were very similar [9].

### Restoring sample representativeness for childhood cognitive ability

**Fig. 2:**
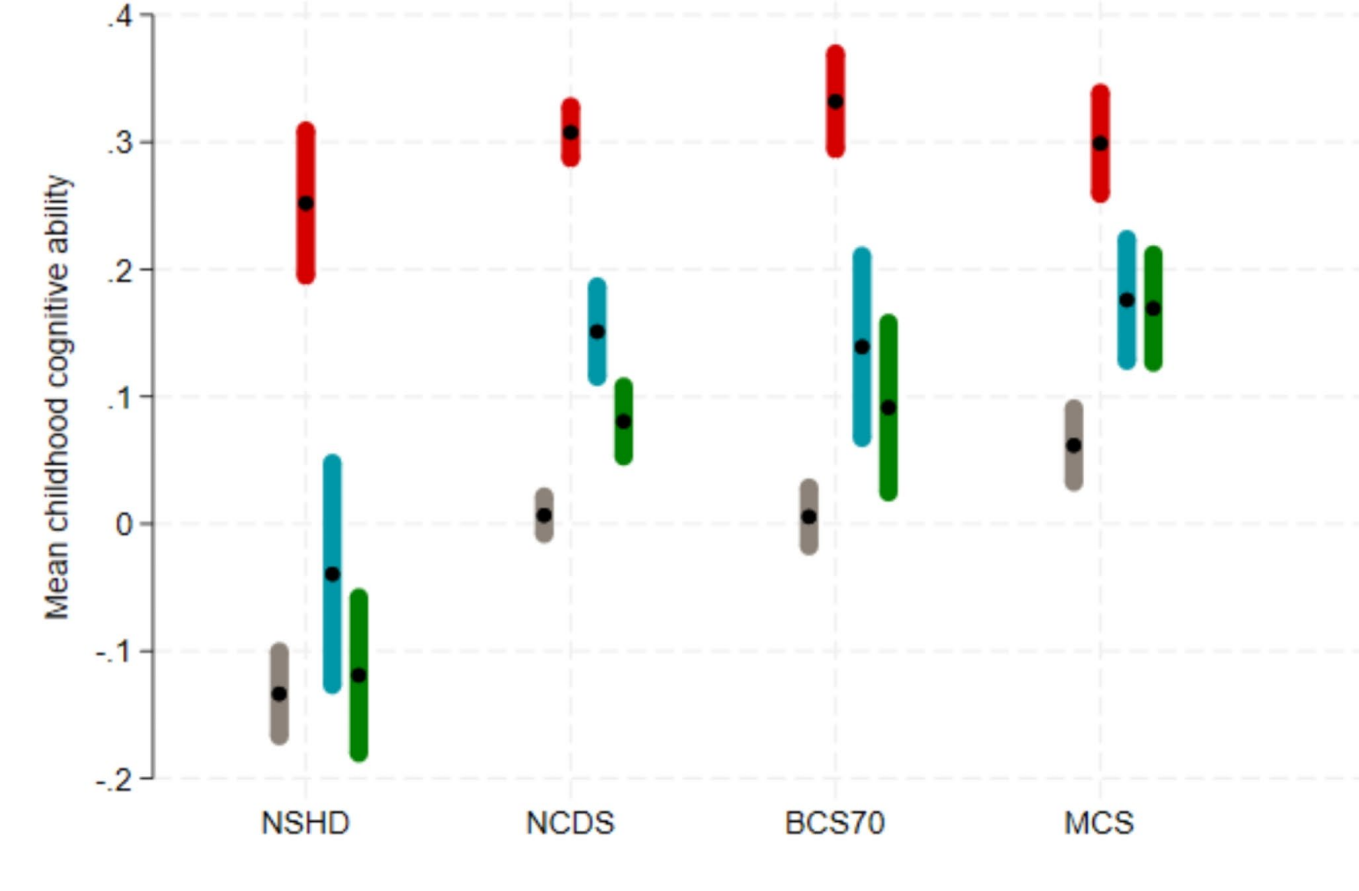
Mean of childhood cognitive ability in each cohort under different estimation approaches to account for non-response in the COVID-19 Wave 1 survey. Grey, first line: using observed baseline data from the whole cohort; red, second line: using observed baseline data from COVID-19 Wave 1 survey respondents only– unweighted (NCDS and BCS70) or using design weight only (NSHD, Next Steps and MCS); blue, third line: using observed baseline data from COVID-19 Wave 1 survey respondents only– weighted using non-response weights (in addition to design weights as appropriate); green, fourth line: using multiple imputation (plus design weight as appropriate). NSHD: National Survey of Health and Development; NCDS: 1958 National Child Development Study; BCS70: 1970 British Cohort Study; MCS: Millennium Cohort Study. Design weights were used in the estimation of means when available (NSHD, MCS) which explains why the mean of the standardised score is not always exactly 0

Considerable bias was also found in childhood cognitive ability, with respondents showing higher means compared to the original sample (Fig. 2 for Wave 1). Non-response weights and MI greatly reduced this bias in all cohorts. While the bias is not fully removed for NCDS, BCS70 and MCS, MI estimates for NSHD show that the bias is near eliminated. Results for Wave 2 and 3 were very similar to Wave 1 [9].

### Sensitivity analyses

Sensitivity analyses showed the newly created non-response weights produced similar results as compared to the original non-response weights from the COVID-19 user guide. For more details see Narayanan at al. [9].

## Discussion

Response rates in these COVID-19 surveys were lower compared to pre-pandemic waves of the same studies, especially for younger cohorts (NSHD: 62–90% versus 84% in 2014/16, NCDS: 54–59% versus 58% in 2013/14, BCS: 40–45% versus 70% in 2016, Next Steps: 20–34% versus 49% in 2015, MCS 24–33% versus 73% in 2018) [8]. Similarly, the Office for National Statistics (ONS) reports decreased response rates for younger participants (0 to 45 years) during the pandemic in other representative surveys [10]. It appears that especially for younger generations, data collected during the pandemic faced increased issues of non-response and thus an increased risk of bias.

We did find bias due to non-response for our chosen examples, with more respondents in the most advantaged social class and with higher mean childhood cognitive ability as compared to the original cohort sample. The application of non-response weights and MI successfully reduced bias in parental social class and childhood cognitive ability, nearly eliminating it for the former. These serve as examples to show how the application of these approaches can reduce bias and increase sample representativeness for the COVID-19 survey waves. Our findings are in agreement with previous work on the effectiveness of non-response weights and MI in the three of these cohorts [4–6].

Making use of COVID-19 surveys embedded within existing longitudinal studies offers a clear advantage over research based solely on COVID-19 samples which lack pre-pandemic data. Our approach enables the reduction of systematic bias, resulting in more robust findings to explore the pandemic’s medium and long-term effects.
